# Experiences of cardiac arrest survivors among young exercisers in Norway: A qualitative study

**DOI:** 10.1016/j.resplu.2022.100293

**Published:** 2022-08-22

**Authors:** Camilla Hardeland, Ann-Chatrin Linqvist Leonardsen, Cecilie Benedicte Isern, Hilde Moseby Berge

**Affiliations:** a Norwegian National Advisory Unit on Prehospital Emergency Medicine (NAKOS), Division of Prehospital Services, Oslo University Hospital, Norway; b Faculty of Health, Welfare and Organization, Østfold University College, Norway; c Department of Anesthesia, Østfold Hospital Trust, Norway; d Faculty of Medicine, Institute of Clinical Medicine, University of Oslo, Norway; e Oslo Sports Trauma Research Center, Department of Sports Medicine, Norwegian School of Sport Sciences, Norway

**Keywords:** Sudden cardiac arrest, Survivors, Experiences, Symptoms, Post-resuscitation follow-up, Qualitative methods

## Abstract

**Aim:**

To explore how young exercisers experience surviving sudden cardiac arrest (SCA), focusing on interpretation of warning signs and experiences with the healthcare system.

**Methods:**

The study had a qualitative design, and data was collected using individual, semi-structured interviews. Inclusion criteria were SCA survivors aged 18–50 years old who reported at least five hours of exercise/week prior to SCA, or who suffered SCA during or ≤60 min after exercise.

**Results:**

18 interviews were performed (4 females), age range 19–49 years old. Analysis identified the themes [1] neglected warning signs, [2] fluctuating between gratitude and criticism and [3] one size does not fit all. When young exercisers experienced symptoms such as fainting, chest pain, arrythmia, shortness of breath and fatigue, these were often ignored by either the participants, healthcare personnel or both. SCA survivors were grateful to the healthcare system and for the efforts made by healthcare personnel, but experienced a mismatch between what patients needed and could utilize, and what they actually received regarding both information and individualised services. Being young exercisers, the participants reported to have individual needs, but treatment and rehabilitation were not adapted and were mainly targeted to rehabilitation of older patients.

**Conclusion:**

Patients and healthcare personnel should be aware of cardiac related symptoms and warning signs for SCA, and these should be properly assessed in the population of young exercisers. SCA survivors need useful and repeated information. The needs of SCA survivors among young exercisers require individualisation of services.

## Introduction

Sudden cardiac arrest (SCA) refer to the sudden cessation of cardiac activity with hemodynamic collapse, typically due to sustained ventricular tachycardia/ventricular fibrillation.^1^ In Norway, overall survival of SCA patients treated by the emergency medical services (EMS) is 14 %, and the median age in this patient population is 68 years.^2^ Survival also relates to the overall health of the patient, and where the SCA occurs; e.g. in a 2019 study, Drezner et al.^3^ found an overall survival after SCA of 48 percent (95 % CI, 40–57 percent) in young athletes during sports, increasing to 89 percent if an on-site automated external defibrillator was used in the resuscitation. Deasy et al.^4^ found higher survival rates in young adults compared to older adults with presumed cardiac aethiology (14.8 % vs 9.0 %, p < 0.001). In a study of symptoms preceding sports-related sudden cardiac death in persons aged 1–49 years, Stormholt et al.^5^ found that up to 74 % of the victims experienced symptoms prior to the cardiac arrest. The main symptoms were syncope, chest pain, palpitations and dizziness. There is a growing body of literature that explore the lived experiences of SCA survivors6., 7., 8., 9., 10. identifying challenges survivors experience such as work-related challenges, impact of social support networks and psychologic, psychic and physical recovery.^7^ In order to meet the needs of patients and provide optimal post-resuscitation follow-up, healthcare professionals need to have specific knowledge about these challenges.

The European Resuscitation Council recommends follow-up of all cardiac arrest survivors three months after hospital discharge providing information and support and screening for cognitive and emotional problems and fatigue.^11^ There is wide variation in the provision of rehabilitation services,^12^ and SCA survivors have reported absence of post-SCA care and a crucial lack of guidance on how to return to and manage daily life.6., 10. However, SCA survivors represent a heterogeneous group of patients, often with unique and complex needs that are inadequately addressed by current treatment recommendations.^13^

Young exercisers are rarely investigated in regards to post-resuscitation follow-up. For this group, a SCA may have a greater impact than in the elderly, due to different life situations and potential challenges such as education, employment and work, income and finance, housing and familiar responsibilities. Consequently, the overall aim of this study was to explore how young exercisers experience surviving a SCA. Specific objectives were to explore^1^ potential warning signs and how these were interpreted by the patients themselves as well as by healthcare personnel, and^2^ experiences with the healthcare system before, during and after the SCA.

## Methods

The study had a qualitative design, using individual semistructured interviews with SCA survivors. The purpose of semistructured interviews are to obtain descriptions of the life world of the interviewee in order to interpret the meaning of the described phenomena.^14^ This is a relevant approach for this study which aims to capture experiences of SCA through the eyes of the patients. The manuscript adheres to the Consolidated criteria for Reporting Qualitative research (COREQ).^15^

### Study design and participants

This descriptive, qualitative study was part of a larger study by the Norwegian Cardiac Arrest Registry aiming to explore incidence, risk factors, aetiology and prognosis for exercise-related versus non-exercise-related SCA in a young Norwegian population. Patients registered in the Norwegian Cardiac Arrest Registry through 2015–2017 suffering from SCA without an obvious external cause in the age group 12–50 years, nationally, were included. Survivors of SCA eligible for inclusion received a questionnaire where we examined relation between the SCA and exercise, exercise-habits and athletic level. We defined exercise as physical activity conducted with the purpose to maintain or improve physical capacity. Study participants were asked permission to be contacted for an interview. For the current study, we invited patients who reported on average at least five hours of exercise/week prior to SCA, and/or who suffered SCA during or ≤60 min after exercise. A purposeful sampling strategy was used, including persons with the highest reported level of training per week first. Interviews were continued until theme saturation.^16^

### Data collection and research team

We conducted in-depth, semistructured interviews in the period September to December 2020. A semistructured interview guide was developed based on suggestions from four user representatives, and discussions among the authors (See Appendix 1). All interviews were conducted individually as video conferences due to the Covid-19 pandemic, using a digital software, Microsoft Teams©. Three of the authors (first, second and last author) were involved in performing interviews. In most interviews two interviewers were present, at least one of the interviewers (first and second author) well experienced with qualitative interviews. To ensure rigour, the first three interviews were performed and discussed by the first and second author together, and in the following interviews either the first or the second author had a leading role, providing the second interviewer the possibility to ask follow-up questions, if required. The first and second author had successive discussions throughout the recruitment period to determine saturation. The audio was taped and transcribed verbatim by an external transcriber, who had signed a non-disclosure agreement.

### Data analysis

Data analysis was performed manually by the authors, using a software program, Hyper Research©, to organise the extensive data material in the analysis process. Data were analysed using thematic analysis as recommended by Braun and Clarke.^17^ This includes the following steps: 1) familiarising yourself with the data through reading and re-reading the transcripts, 2) generating initial codes, 3) searching for themes across all interviews, 4) reviewing, defining and naming themes, and 5) producing the report. The first and second author read and re-read the transcripts individually and conducted the initial coding of all interviews together. Identification of initial themes were done both during the coding process and in later meetings. This enabled a thorough and iterative analysis process where all codes and themes were continuously discussed, and relevant quotes identified. After the first and second author had made suggestions for initial themes and subthemes, these were discussed with all authors. A consensus about the interpretation, themes and subthemes was reached among all authors.

Two of the authors were medical doctors involved in the design and data collection for the Norwegian Cardiac Arrest Registry study, the other two authors were registered nurses experienced with emergency care and SCA. This made us aware of discourses, impressions and experiences that could possible impact the interpretations. Hence, a method of reflexivity^18^ was applied throughout the analytic process. This included critical reflection on the research process, both by discussions amongst the authors and through writing reflexivity notes before and after each interview. These notes were consulted throughout the analysis.

### Ethical considerations

The study was approved by the Regional ethics committee (ref. no 2016/671) and the local data protection authority at Oslo University Hospital (ref. no 17/18457). The study was guided by the Declaration of Helsinki,^19^ and based on willing, informed consent to participate, the right to withdraw at any time without any negative consequences, and on anonymity/confidentiality.

## Results

Of an initial pool of 31 eligible participants, data saturation was reached after 18 interviews and further data collection was ceased. Their median age at SCA was 43 years (mean 37, range 19–49), four were females. Median time since SCA was 46 months (mean 46, range 34–68 months). Median time from SCA to responding to questionnaire about exercise-habits was 22 months (mean 22, range 12–40 months). In total, 12 participants met the criteria of a minimum of five hours of exercise per week on average, while 11 participants met the criteria of SCA occurred during or <60 min after physical activity. Of these, six reported less than five hours of exercise per week. The interviews lasted from 24 to 70 min.

Through analysis we identified the following themes: [1] neglected warning signs, [2] fluctuating between gratitude and criticism and [3] one size does not fit all.

### Neglected warning signs

Most of the participants reported several warnings signs that in retrospect could be related to the SCA, but neither participants nor healthcare personnel considered them as such. One of the participants reported: “*I fainted, and the ambulance was called. But, nobody took an ECG because they believed I had a head contusion*”.

Many of the participants had a sensation of fatigue and felt exhausted both over a period of time prior to the arrest, but also on the day of the incident. One of the participants even reported to be hospitalised twice due to fatigue, but given no explanations. Some had experienced abnormal breathlessness during physical activity, others had felt they were in great physical shape when exercising, but exhausted at rest, yet some that extensive training did not provide expected results.

Different forms of arrhythmia before the SCA were reported, such as bradycardia, tachycardia, irregular pulse and palpitations. Ten of the participants had various symptoms such as dizziness, near-syncope or syncope, central chest or back pain. In most cases, they did not contact healthcare services due to these symptoms, as they were considered caused by stress, muscle tensions, sleep deprivation, and in some cases, due to having small children. As one of the participants said: “*There were so many reasons for me being tired and exhausted…”.* When in contact with healthcare personnel, the symptoms were also dismissed, and not assessed as cardiac-related. The explanations could be that the participant was «too young», «too healthy» or «too fit» to need further investigations, or that the symptoms were caused by allergy, asthma or anaemia.

### Fluctuating between gratitude and criticism

Most of the participants were grateful for the healthcare services they had received and reported a positive impression of the emergency medical dispatch system. They referred to the system as «professional», «a safety net», that «worked 110 percent», and “providing essential guidance to the bystanders calling for help”. This was based on second-hand information given from relatives or healthcare personnel, since participants themselves had no or little recall from the SCA event.

Likewise, most reported to have been in a coma or sleeping the first days of the hospital stay, and memories were brief. Still, most praised the healthcare personnel for being attentive and nice. Only two of them could remember whether the information they received during the hospitalisation was sufficient and met their needs. Four of the participants experienced that healthcare personnel were unaware of the amnesia, expecting the patients to remember information. One of them stated: “*Several doctors came to see me and talked to me as if I knew them, but I really didn’t*”.

Six of the participants emphasised the need for repeated oral and written information. 14 of the participants received an Implantable Cardioverter Defibrillator (ICD), but only four of them remembered to have received sufficient knowledge about the consequences of having an ICD and how to handle it after hospital discharge. This resulted in one participant getting contractures in the arm, because he didn’t dare to move it. Another participant said: “*I have had episodes where the ICD started alarming, and I got really scared and worried because I hadn’t got any information about that*”. In addition, few of them knew what to do if the ICD gave a shock. In one instance one of the participants were skiing in the mountains on a sleepover-trip without mobile cell-phone coverage when getting a shock, and did not know whether or where to call for help or what to do.

Hence, the criticism was related to a mismatch of information between what participants experienced to need and what they actually received.

### One size does not fit all

Participants experienced that healthcare services were not adapted to their situation, being young exercisers, several of them in family settings with small children. When in hospital, several of them had negative experiences sharing a room with older patients with other symptoms and challenges. One of them stated: “*It was difficult to share a room with demented patients who peed in the corners, hacking and hawking*”, and “*My kids wanted to come visit, but they thought it was extremely frightening and were scared to death*”.

The participants reported none, or a wide range of rehabilitation or follow-ups after the SCA, such as a three weeks rehabilitation program, training programs for cardiac patients, follow-up at the hospital or the general practitioner. Some did not remember whether they had been offered rehabilitation. The ones with most severe complications in retrospect, such as speaking difficulties, received ergotherapy or neuropsychological training, but these services seemed not to be standardised.

All of the participants who had attended a rehabilitation program reported that other patients were mainly older, and that the programs seemed age-adjusted to them. One of them stated: “*It was much focus on how to stop using cream in the coffee and stop eating cookies*”. The physical exercises were mainly too easy, compared to their capacity. In contrast, one physician told the participant he could resume regular exercise immediately after hospital discharge. However, it turned out the physician meant walks around the block, while the participant perceived he could go directly to the gym.

One of the participants also talked about having to combine rehabilitation with the «daily program» at home, having to deliver children in the kindergarten, cook or tidy the house to avoid extra burden on the partner. Three of the participants stated that they would have preferred rehabilitation groups if they were restricted to persons on their own age.

## Discussion

This study explores young exercisers’ experiences with surviving SCA; before the event including warning signs, from the hospital stay and with rehabilitation services after discharge. Our results show that, in this population, warning symptoms are not being suspected as cardiac-related, and there is a mismatch between healthcare services and what the patients need after SCA.

Even though most of the participants had symptoms such as feeling exhausted, dizziness, near-syncope, central chest- or back pain, or arrythmia, these were not suspected to be cardiac related or even in need of healthcare services. This is supported by e.g. Lau et al.,^20^ who reported that survivors expressed disbelief, guilt and surprise that a SCA does not always have obvious warning signs. Participants in our study did not necessarily contact healthcare services, or if they did, they felt eased that there was nothing to worry about. This is in contrast to Forslund et al.^21^ who found that participants experienced various somatic phenomena some time before the SCA. Ketilsdottir et al.^22^ reported that all participants experienced forewarning symptoms such as heartburn or decreased endurance before the SCA. Participants thought it was difficult to contact healthcare services without an obvious reason.

Several studies have focused on identifying cardiac-related warning signs to prevent SCA.23., 24., 25., 26. Some symptoms are acknowledged in clinical practice as related to cardiac disease at risk of SCA, such as chest pain with or without referred pain, syncope, and dyspnoea.23., 24., 25., 26. However, symptoms can be nonspecific, and there is likely to be significant overlap between those that precede acute myocardial infarction, other cardiac disease, or even non-cardiac conditions.^24^ In a study of 839 SCA patients,^26^ symptoms recurred in 51 percent during the 24 hours preceding the SCA, still only 19 percent called emergency medical services. In the current study of young exercisers, the findings indicate that symptoms are not taken seriously as is the case in other SCA populations as well. SCA in athletes is a recurrent phenomenon,27., 28., 29., 30. but to date, researchers have not been able to develop an efficient method of preventing sudden cardiac death in athletes.^27^

Further, our results indicate that these SCA survivors need individualised services both in hospital and during rehabilitation. In addition, they reported a need for repeated information. Wagner et al.^31^ found a lack of support from the health care services in the transition from hospital to daily life. Other studies have emphasised the challenges experienced by SCA patients after discharge from hospital, feeling vulnerable and abandoned and continuously seeking information.21., 32., 33.

To our knowledge, there are no recommendations for follow-up after hospitalisation for SCA in Norway. A survey study in Sweden revealed that although local guidelines for follow-up exist at some hospitals, they are not uniformly applied or explicit.^34^ According to the American Heart Association (AHA)^13^ a lack of standardisation of patient-centred assessment and reliable provision of resources, discharge from hospital will stay fragmented.

A 2015 systematic review of studies of the outcomes reported in SCA trials demonstrated inconsistencies across the complete cardiac arrest patient journey.^35^ The authors conclude that assessments focusing on survival, body structure and -function are important to understand the clinical impact of SCA, but less helpful when seeking to understand the lived experiences of survivors.

### Strength and limitations

Within the qualitative research design lies the limitation that findings are not generalisable. Due to the heterogeneity of SCA survivors, and the fact that we included only four women, our findings may not be transferable to other settings and populations. There is potentially a recall bias in participants’ reports of hours of exercise per week, due to the mean time of 22 months from SCA to filling out the questionnaire about exercise-habits.

The research group consists of two medical doctors and two registered nurses, all female. We used a method of reflexivity, and all researchers actively engaged in the analytic process, which increase the trustworthiness of the interpretations.

We have only interpreted the participants’ symptoms as “warning signs” before the SCA. We cannot state for sure that this is the case. However, the symptoms reported by participants in our study correlates with symptoms reported in other studies25., 26., 27. which increase the validity of our findings.

## Conclusion

This study is the first to explore young exercisers experiences with SCA; before the event, from the hospital stay and with rehabilitation services after discharge. Results underline the importance of identifying symptoms and further assess these in this population. Following SCA, survivors need repeated information to enable them to get on with their lives. Individualised services include a whole-person approach, taking the patients’ life-situation into consideration. Furthermore, the needs of SCA survivors among young exercisers require individualisation of services. We suggest that recommendations for post-SCA care are developed and standardised.

## Declaration of interests

The authors declare that they have no known competing financial interests or personal relationships that could have appeared to influence the work reported in this paper.

## CRediT authorship contribution statement

**Camilla Hardeland:** Conceptualization, Methodology, Validation, Formal analysis, Investigation, Data curation, Writing – original draft, Writing – review & editing. **Ann-Chatrin Linqvist Leonardsen:** Conceptualization, Methodology, Validation, Formal analysis, Investigation, Data curation, Writing – original draft, Writing – review & editing. **Cecilie Benedicte Isern:** Conceptualization, Methodology, Validation, Formal analysis, Investigation, Data curation, Writing – original draft, Writing – review & editing. **Hilde Moseby Berge:** Conceptualization, Methodology, Validation, Formal analysis, Investigation, Data curation, Writing – original draft, Writing – review & editing.
