# Supplementary material for: Experiences of cardiac arrest survivors among young exercisers in Norway: A qualitative study
Source: Resusc Plus. 2022 Aug 22;11:100293. doi: 10.1016/j.resplu.2022.100293 (PMC9424599; doi:10.1016/j.resplu.2022.100293)
Supplement: Supplementary data 1 [file mmc1.docx]

Appendix 1 Interview guide

­­­­­­­­­­­­­­­­­­­­­­­­­__________________________________________________________________________________

Welcome and introduction to the interview. Information about confidentiality, anonymisation and the possibility of withdrawing from the study.

(Background information: from questionnaire)

**Prior to SCA:**

● Can you say something about your activity level before the cardiac arrest? (Follow-up question: were you physically active? How / how often?)

● Did you have any pain or discomfort that you later think may have had something to do with the cardiac arrest? (If so: how did you relate to these ailments? Were you in contact with a doctor? Did you find out what the ailments came from? Did you hesitate to go to the health service again?)

**Related to the incident:**

If you think back to the day you had a cardiac arrest:

● Do you remember any of the things that happened when you had a cardiac arrest? (Follow-up: Do you remember what you did just before you had a cardiac arrest? Do you remember if you had any special symptoms?)

● Have you received more information about what happened afterwards? (Follow-up question: Can you elaborate? Who told you this? Did you have to request information yourself? Have you been in contact with anyone who was present during the incident?)

**Related to the time after cardiac arrest:**

● Do you remember anything from your hospital stay? (Follow-up question: How did you experience the meeting with health professionals? What kind of information did you receive? Was there anything you wanted information about that you did not receive?)

● Have you received any kind of information, follow-up or support from the health service after the incident? (Follow-up: The hospital? GP? Other?)

● Do you know the cause of your cardiac arrest? (Can you elaborate?)

● Is it other than the health service you have experienced receiving follow-up or support from? (Follow-up: Can you elaborate on this? Do you feel that you have received any support from family / friends / coaches / training mates / employer after the cardiac arrest? Does anyone call or contact you and ask how you are?)

● How has cardiac arrest affected you in everyday life? (Follow-up question: Are you studying? Are you at work? Has anything changed since then? Has this had any financial consequences for you? Do you have any problems that you relate to cardiac arrest? Can you do what you did before? Elaborate? Are you worried that this will happen again?)

● Can you say something about your activity level after the event? (Follow-up question: are you physically active? How / how often?)

● Do you experience that someone around you (family, friends, others) treats you differently after the incident?

● Have you received good information about the ICD and what to do if it hits?

● Have you needed to talk to someone who has been in the same situation as you after the cardiac arrest? (Young and fit survivors of cardiac arrest)

● Have you read journal notes from the incident afterwards?

**Closing:**

● Is there anything you would like to highlight from your experiences around cardiac arrest that has been particularly good / positive?

● Is there anything you would like to highlight from your experiences around cardiac arrest that has been particularly negative?

● Is there anything I have not asked that you would like to add?

Thanks to the participant.
